# In Vitro Evaluation of Antimicrobial Peptide Tridecaptin M in Combination with Other Antibiotics against Multidrug Resistant *Acinetobacter baumannii*

**DOI:** 10.3390/molecules25143255

**Published:** 2020-07-17

**Authors:** Manoj Jangra, Vrushali Raka, Hemraj Nandanwar

**Affiliations:** 1 Clinical Microbiology & Bioactive Screening Laboratory, CSIR-Institute of Microbial Technology, Chandigarh 160 036, India; manojjangra91@gmail.com (M.J.); vrushali.raka15@gmail.com (V.R.); 2 Academy of Scientific and Innovative Research (AcSIR), Ghaziabad, Uttar Pradesh 201 002, India

**Keywords:** Tridecaptin M, *Acinetobacter baumannii*, combination therapy, Gram-negative bacteria, antibiotic-resistance, persisters

## Abstract

The rapid emergence of antimicrobial resistance in *Acinetobacter baumannii* coupled with the dried pipeline of novel treatments has driven the search for new therapeutic modalities. Gram-negative bacteria have an extra outer membrane that serves as a permeability barrier for various hydrophobic and/or large compounds. One of the popular approaches to tackle this penetration barrier is use of potentiators or adjuvants in combination with traditional antibiotics. This study reports the in vitro potential of an antimicrobial peptide tridecaptin M in combination with other antibiotics against different strains of *A. baumannii*. Tridecaptin M sensitized the bacteria to rifampicin, vancomycin, and ceftazidime. Further, we observed that a tridecaptin M and rifampicin combination killed the bacteria completely in 4 h in an ex vivo blood infection model and was superior to rifampicin monotherapy. The study also found that concomitant administration of both compounds is not necessary to achieve the antimicrobial effect. Bacteria pre-treated with tridecaptin M (for 2–4 h) followed by exposure to rifampicin showed similar killing as obtained for combined treatment. Additionally, this combination hampered the survival of persister development in comparison to rifampicin alone. These findings encourage the future investigation of this combination to treat severe infections caused by extremely drug-resistant *A. baumannii*.

## 1. Introduction

Gram-negative bacteria (GNB) such as *Escherichia coli*, *Klebsiella pneumoniae*, *Pseudomonas aeruginosa*, and *Acinetobacter baumannii* can thwart the detrimental effect of most of the antibiotics, due to the permeability barrier posed by the outer membrane [1,2]. Only a limited number of drugs can cross this barrier and exert antimicrobial activity in these pathogens. Examples of such compounds include carbapenems [3], polymyxins [4], and other peptide antibiotics, such as octapeptins [5] and the recently discovered darobactin [6]. The emerging resistance to even last-line antibiotics in Gram-negative pathogens has led to a global health crisis. The World Health Organization has classified carbapenem-resistant GNB as pathogens of critical priority which urgently require the development of new therapeutics [7]. The topmost pathogen, *A. baumannii* causes several life-threating nosocomial infections. The mortality rate of *A. baumannii* infections in intensive care units may go up to 70% [8]. 

At present, only a few treatment options are left to treat drug-resistant *A. baumannii* infections [9,10]. Tetracyclines such as tigecycline or eravacycline and aminoglycosides (amikacin) are used to treat *A. baumannii* infection. However, tigecycline suffers from pharmacokinetic issues, and amikacin has nephrotoxicity and high resistance rates. The preferred antibiotics for susceptible bacteria are usually cephalosporins or carbapenems [8]. However, resistance to these classes is growing expeditiously [11,12]. Colistin is now the antibiotic of choice for the treatment of carbapenem-resistant *A. baumannii* (CRAB) infections, despite having severe nephrotoxicity [13,14]. The appearance of colistin-resistance, however, in CRAB or other GNB is of grave concern [15,16,17,18]. There is a significant gap in the pipeline of new antibacterial agents that target CRAB currently in development [19,20]. Moreover, most of the agents are analogues of the existing classes of antibiotics, and the lion’s share of the pipeline (against CRAB) is occupied by β-lactam antibiotics and β-lactamase inhibitors. The rapid emergence of resistance to these existing classes following their use in the market calls for the development of novel therapeutics that target unique enzymes or pathways in these bacteria.

While the discovery of novel antimicrobial agents is critical and of vital importance, the recent approaches applied by several researchers or healthcare professionals to tackle drug-resistant *A. baumannii* include drug-repurposing or combination therapy [21,22,23]. The traditional combination therapy involved the use of two antimicrobial agents as an empirical therapy because the infecting organism may be susceptible to at least one of the agents. However, the definitive combination therapy is somewhat different, and it is useful in certain circumstances, such as for patients in intensive care units where single antibiotic treatment fails [24]. The very advantage of combination therapy is hitting multiple targets at the same time. This strategy makes bacteria less invincible to the development of acquired resistance [25]. The combination therapy generally includes two antibiotics which may or may not have synergy between them [24] (a combination of more than two drugs generates further complexity in the treatment and is not the scope of the present study). Several reports have demonstrated the synergy among the different antibiotics against multidrug-resistant (MDR), extremely drug-resistant (XDR), or pandrug-resistant (PDR) *A. baumannii* infections [26,27,28,29]. In most combinations, colistin was used as one of the components. However, there are a few limitations within the existing combination treatment regimens. Resistance is already reported for colistin or other antibiotics used in combination, and secondly, these combinations are not proven statistically superior to monotherapy [11]. There is a pressing need to develop superior combination therapy to treat severe infections caused by carbapenem- and colistin-resistant *A. baumannii* where treatment with a single antibiotic is not efficacious. 

**Figure 1:**
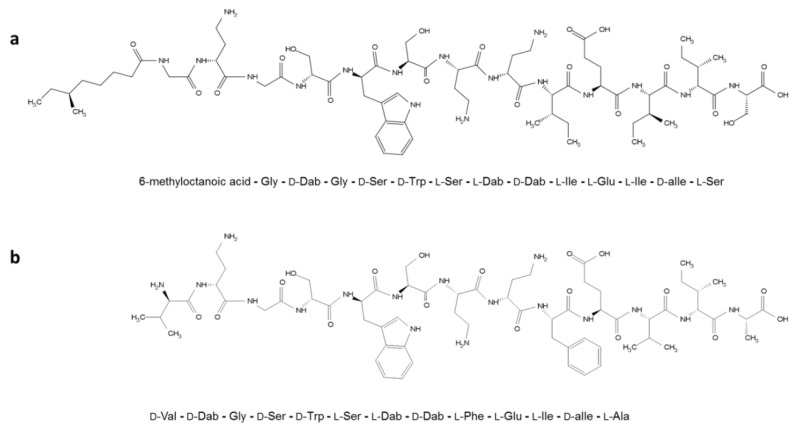
Chemical structures and amino acid sequences of (**a**) tridecaptin M and (**b**) unacylated tridecaptin A_1_. The lipid moiety in natural tridecaptin A_1_ is 6-methyl-3-hydroxy octanoic acid.

Several cationic antimicrobial peptides have been reported to potentiate the activity of traditional antibiotics [30,31,32,33,34]. Tridecaptins are a class of lipopeptide antibiotics that retain activity against multidrug-resistant Gram-negative bacteria. The main members of this class include tridecaptin A [35], tridecaptin B [36], and tridecaptin M [37]. Recently, unacylated tridecaptin A_1_ (without the lipid moiety; Figure 1), though ineffective itself, was reported to potentiate (or reduce the minimum inhibitory concentration of) Gram-positive antibiotics in Gram-negative bacteria including *Enterobacteriaceae* and *A. baumannii* [38]. Tridecaptins have very low intrinsic activity against *A. baumannii* [36,37], but they could still permeabilize the outer membrane very efficiently [39]. The naturally occurring tridecaptins are active against *Enterobacteriaceae* but have not been investigated for their potential in combination therapy. Tridecaptin M is already under preclinical development for XDR *Enterobacteriaceae* [37] (Figure 1); therefore, the focus of the present study was to evaluate the synergistic activity of tridecaptin M (at the concentration to be used for *Enterobacteriaceae*) with other antibiotics against *A. baumannii*. Further, the study tested the potentiation of rifampicin in different strains of *A. baumannii* and also investigated the development of a persister population in the presence of combination therapy. This combination might prove effective in treating XDR or PDR *A. baumannii* infections. 

## 2. Results

### 2.1. Tridecaptin M Disrupts the Outer Membrane Effectively

**Figure 2:**
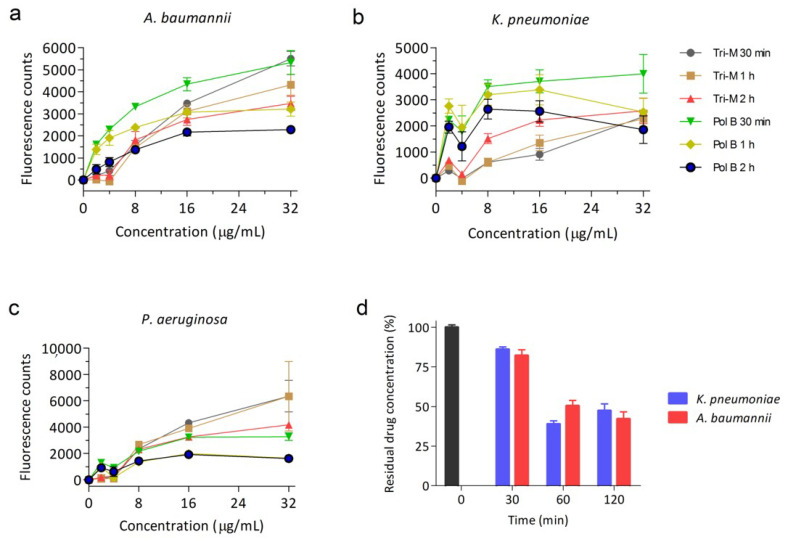
Tridecaptin M shows outer membrane disruption in all Gram-negative bacteria. (**a**) *N*-phenyl-1-naphthylamin (NPN) assay with *A. baumannii*. (**b**) NPN assay in *K. pneumoniae*. (**c**) NPN assay in *P. aeruginosa*. The data plotted are mean values of three biological replicates. Bars denote the standard deviation. Tri-M—tridecaptin M and Pol B—polymyxin B. (**d**) Binding kinetics of tridecaptin to bacteria determined by reversed-phase high pressure liquid chromatography (RP-HPLC) using area under the curve. The concentration of tridecaptin M was 16 µg/mL. The data plotted are the mean of three replicates and representative of two biological repeats. Bars denote standard deviation.

Tridecaptin M was tested to assess the effect on the outer membrane of different Gram-negative bacteria. We previously showed that tridecaptin M has weak antimicrobial activity in *A. baumannii* and was not active against *P. aeruginosa* [39]. However, similar to previously reported results, we observed strong permeabilization of the outer membrane in a concentration-dependent manner in all the pathogens as denoted by the increase in fluorescence intensity of *N*-phenyl-1-naphthylamin (NPN) dye (Figure 2a–c). NPN is a hydrophobic dye which exhibits negligible or low fluorescence in aqueous conditions (bacterial surrounding), whereas an increase in fluorescence indicates the localization of NPN in the periplasm and phospholipid membrane, suggesting disruption of the outer membrane [40]. In *A. baumannii*, the fluorescence was similar to that caused by polymyxin B. *P. aeruginosa* cells were even more prone to outer membrane disruption, and the effect was much higher than in polymyxin B. It was surprising to see that in *K. pneumoniae*, though being most sensitive to tridecaptins, fluorescence increased to the least. While tridecaptin M showed differences in fluorescence of NPN, the binding affinity of the compound to bacteria was similar for all of them (data for *P. aeruginosa* not shown here, but binding kinetics were similar) (Figure 2d). These results indicate that binding of tridecaptins and permeabilization of the outer membrane are not sufficient for the killing of bacteria in case of *A. baumannii* and *P. aeruginosa*. 

### 2.2. Synergy of Tridecaptin M with Various Antibiotics in A. baumannii 

**Figure 3:**
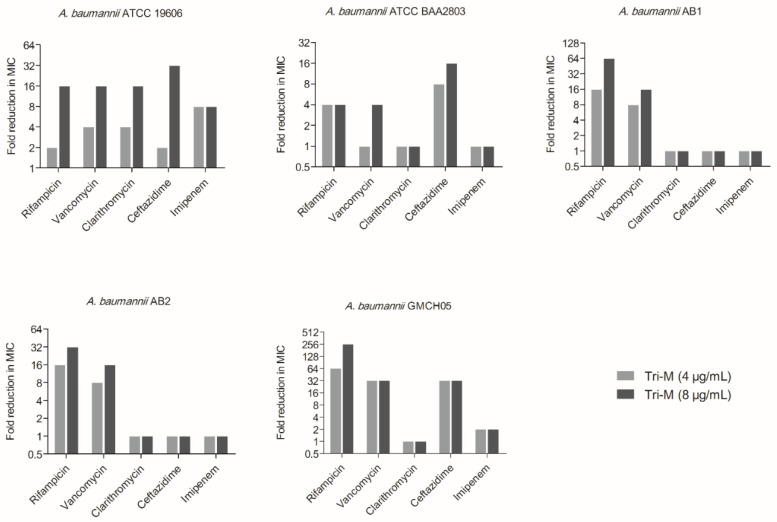
Synergy of tridecaptin M with other antibiotics in *A. baumannii* strains at two different concentrations. The values presented here are the mode of three independent experiments.

With the disruption of the outer membrane, we wondered whether tridecaptin M could potentiate the activity of other antibiotics (those lacking efficacy due to penetration problems) in Gram-negative bacteria. For preliminary testing, a checkerboard assay of tridecaptin M with rifampicin or vancomycin, was performed. The rationale for choosing these two classes of antibiotics was that they were previously reported for potentiation against Gram-negative bacteria in the presence of unacylated tridecaptin A_1_ [38]. The unacylated tridecaptin A_1_ did not have inhibitory activity itself up to 100 µM, but at sub-inhibitory concentration, it could reduce the concentration of these antibiotics to kill the bacteria. We were interested in studying the synergy of a natural tridecaptin variant. As shown in Table 1, the synergy was observed in *A. baumannii* only. This was in contrast to unacylated tridecaptin A_1_ which showed synergy in *Enterobacteriaceae* as well [38]. Moreover, no synergy was obtained where the tridecaptin M concentration was below 4 µg/mL. At 0.25× of its MIC (minimum inhibitory concentration, i.e., 8 µg/mL), tridecaptin M reduced the MIC of vancomycin and rifampicin against *A. baumannii* by 16-fold which was comparable to results obtained for unacylated tridecaptin A_1_ by Cochrane et al. [38]. Nevertheless, it is well understood that no single antibiotic regimen is universally effective against all types of bacterial infections; we next studied the potentiation of various antibiotics in five different MDR strains of *A. baumannii*. The strains included one fully characterized clinical strain of the American Type Culture Collection (ATCC) special collection, and three of them were isolated from hospital infections in India. Tridecaptin M displayed synergy with all five classes of antibiotics in *A. baumannii* ATCC 19606; with rifampicin, vancomycin, and ceftazidime in ATCC 2803 and GMCH05; and with rifampicin and vancomycin in AB1 and AB2 strains (Table 2). The results suggested that the synergistic activity of antibiotics was strain specific. Notably, the combination of rifampicin or vancomycin with tridecaptin M demonstrated synergy in all the strains tested and their MICs were reduced by a maximum of 256- and 32-fold, respectively, when used at 8 µg/mL (Figure 3).

### 2.3. Tridecaptin M and Rifampicin Combination Shows Efficacy in Rabbit Blood

**Figure 4:**
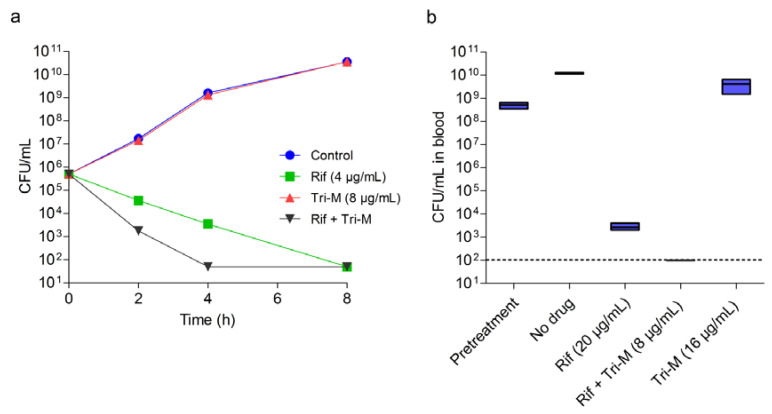
In vitro efficacy of tridecaptin M and rifampicin combination against *A. baumannii* ATCC 19606. (**a**) Killing of bacteria in nutrient-rich medium in the presence of antibiotics alone or in combination. The data are plotted as the mean of three replicates. Bars indicate the standard deviation. The experiment is representative of two biological repeats. (**b**) Killing of bacteria in rabbit blood after 4 h treatment. The data represent mean values of three replicates with standard deviation. The dotted line represents the limit of detection.

Tridecaptin M in combination with rifampicin inhibited four out of five strains (80%) when used at ≤8 µg/mL. In the time-kill experiment using a nutrient-rich medium, this combination killed the bacteria in 4 h, faster than monotherapy (Figure 4a). Nevertheless, the therapeutic efficacy in artificial media usually does not translate to in vivo systems. The stability and plasma binding could interfere significantly with the efficacy of antibiotics [41,42]. To identify the in vivo potential of rifampicin and tridecaptin M combination, we used an ex vivo blood infection model to mimic an in vivo environment where rabbit blood was inoculated with *A. baumannii* (~10^9^ colony forming unit (CFU)/mL). Tridecaptin alone at 16 µg/mL had no effect on bacterial growth (Figure 4b), whereas the combination killed the bacteria below the detection limit in 4 h. The combination (rifampicin, 20 µg/mL and tridecaptin M, 8 µg/mL) reduced ~1.5 logs CFU more than the rifampicin monotherapy.

### 2.4. Concomitant Administration of Both Antibiotics is not Necessary for the Potentiation 

**Figure 5:**
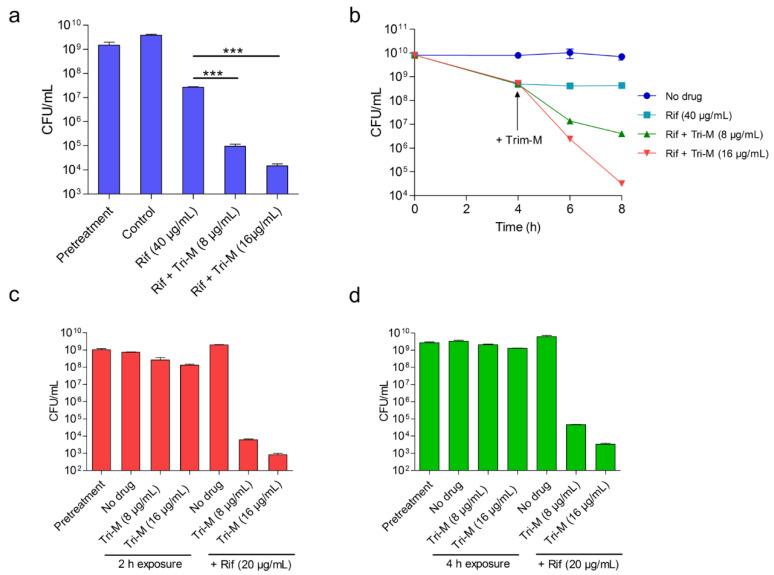
Time-lag kinetics of tridecaptin M and rifampicin combination to study the effect on bacterial killing. (**a**) The late-exponential phase bacteria were exposed with rifampicin or tridecaptin M alone and in combination. In the combination panel, both antibiotics were added at the same time. The significance was calculated using the paired Student’s *t*-test with two-tailed distribution, *** denotes *p* < 0.0001. (**b**) The bacteria were initially incubated with rifampicin, and then tridecaptin M was added. (**c**,**d**) The bacteria were pre-treated with tridecaptin M for 2 h (**c**) or 4 h (**d**), and then tridecaptin was removed by centrifugation followed by the addition of rifampicin. In all the experiments, the data plotted are mean values of three replicates, and bars denote the standard deviation. The experiments are representatives of two biological replicates. In the graphs, the pretreatment bar denotes the initial CFU load before treatment with any of the antibiotics.

To rule out the possibility that both the compounds do not interact with each other and make a complex outside the bacterial surrounding, we treated the bacteria with the compounds at different times and checked the differences in antimicrobial activity. Figure 5a shows the effect on bacterial killing when both antibiotics were added to bacteria at the same time. In 4 h, approximately 5 logs CFU were reduced when rifampicin and tridecaptin M were used at 40 µg/mL and 16 µg/mL, respectively, which was >3 logs CFU greater than the rifampicin monotherapy. When bacteria were initially incubated with rifampicin for 4 h and subsequently treated with tridecaptin M, similar results were obtained (Figure 5b). In the third experiment, bacteria were pre-treated with tridecaptin M and then the effect of the compound was removed followed by the addition of rifampicin (Figure 5c,d). We observed a similar reduction in bacterial load. Of note, rifampicin killed a higher number of bacteria when they were pre-treated with tridecaptin M for 2 h, compared to the 4 h treatment. These results together suggested that the overall efficiency of the combination was independent of the time of their administration (no significant effect up to a time lag of 4 h).

### 2.5. Effect of Combination Therapy on Persister Survival 

**Figure 6:**
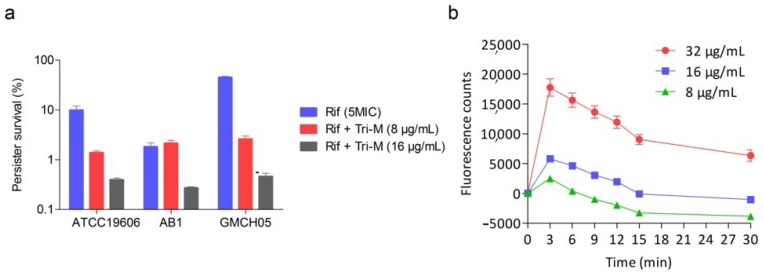
Effect of combination therapy on persisters’ survival. (**a**) The survival of bacterial persisters after 4 h treatment with rifampicin or tridecaptin M alone and in combination. The persisters were confirmed by determining the minimum inhibitory concentration (MIC) of two representatives after subculturing on a fresh agar plate containing no antibiotics. The MIC values were similar, indicating that persisters were not resistant to antibiotics. (**b**) NPN assay with stationary-phase bacteria indicating the permeabilization of persisters with tridecaptin M at different concentrations. Data plotted are mean values of three replicates. Bars denote the standard deviation.

Persisters are sub-populations of cells that are tolerant to antibiotics but not resistant to them [43]. They cause a relapse of infection and are also responsible for treatment failure in many cases. We sought to study the survival of persister populations of three strains of *A. baumannii* in the presence of combination therapy and compared the results with rifampicin monotherapy. Persisters were confirmed by spread plating the culture on an antibiotic-free plate and then two representatives from each plate were studied for MIC determination. The persisters showed no change in MICs for rifampicin or its combination with tridecaptin M, indicating that they were drug-tolerant, not resistant. As depicted in Figure 6a, rifampicin alone had a very high propensity for persisters formation and approximately 2–50% of the bacterial population was persisters. In contrast to this, the combination reduced the survival of persisters, and the survival rate was less than 0.5% in all the tested strains when tridecaptin M concentration was 16 µg/mL. If we compare the reduction in bacterial load in the persister assay with that observed in the late exponential-phase population of bacteria (Figure 5), the combination had less killing activity. For instance, in the persister assay, the reduction was between 2 to 3 logs CFU, whereas against the exponentially growing population, the combination exhibited >5 log reduction. To check whether this difference was due to the inability of permeabilization or non-binding of tridecaptin M to stationary-phase bacteria, we studied the change in fluorescence of NPN using stationary-phase cells. Tridecaptin M showed similar permeabilization of these cells (Figure 6b). Moreover, the binding kinetics was also unaffected due to this difference in the growth phase (data not shown). These findings indicate that the difference in the killing of these two different populations of cells may be due to the low efficacy of rifampicin against the slow-growing cells. Nevertheless, the combination therapy hampered the persisters’ development significantly. 

## 3. Discussion

In this study, we investigated the potentiation of various antibiotics in the presence of a natural tridecaptin variant, i.e., tridecaptin M. Recently, unacylated tridecaptin A_1_ was reported to express synergy with various antimicrobial agents, of which rifampicin and vancomycin were the most modulated ones [38]. The rationale for using a natural tridecaptin molecule was that this molecule is now under preclinical development to treat XDR *Enterobacteriaceae* [37], and we postulated whether this molecule could be used in combination therapy (at the same dose) against other Gram-negative bacteria to increase its antimicrobial spectrum. Moreover, the chemical synthesis of unacylated tridecaptin would cost more compared to fermented product. While screening this compound initially for synergy studies, we took rifampicin and vancomycin to test if the potentiation was due to deacylation of tridecaptin A_1_ because the natural variant was not studied in combination. 

Tridecaptin M demonstrated synergistic activity against *A. baumannii* only, in contrast to unacylated tridecaptin A, which displayed potentiation in *K. pneumoniae* or *E. coli* as well [38]. This was presumably due to the concentration of tridecaptins used in synergy experiments. We initially thought that the concentration of tridecaptins should cross a threshold value at which the synergistic effect appears. For instance, tridecaptin M showed the best synergy against *A. baumannii* when used at 8 µg/mL (MIC 32 µg/mL), and this concentration cannot be used in *K. pneumoniae* or *E. coli* because at this concentration, the antibiotic itself is lethal to the bacteria (MIC 4 µg/mL). On the other hand, the unacylated version was ineffective at this concentration but permeabilized the cells. Hence, it may be concluded that the lipid moiety has no effect on permeabilization and/or probably on potentiation. 

Surprisingly, *P. aeruginosa* showed a greater degree of outer membrane disruption at 8 and 16 µg/mL as shown by the increased fluorescence of NPN dye, but no synergy was obtained. Based on these results, it may be concluded that permeabilization of the outer membrane by tridecaptins may be essential but not the sole factor to drive the sensitization of bacteria to other antibiotics. The strain-specificity of tridecaptin M synergy towards *A. baumannii* may be due to the presence of an altered outer-membrane in this pathogen; most of the clinical strains have lipooligosaccharide (LOS) instead of lipopolysaccharide (LPS) [44]. This compound might cause some modifications to the bacterial cellular components which are crucial for synergy as reported for one of the compounds recently [45]. Additionally, polymyxin and serotonin reuptake inhibitor sertraline combination was shown to affect membrane biogenesis [46]. The exact mechanism responsible for this synergy obtained with tridecaptin M needs further investigation and is beyond the scope of this study. 

The study next focused on *A. baumannii* only and the synergy was examined in five different strains with various antibiotics. Tridecaptin M showed the best synergy with rifampicin and vancomycin, but the concentration of vancomycin required to achieve the therapeutic efficacy was still high (8 or more than 8 µg/mL; vancomycin MIC is 0.5–2 µg/mL for susceptible Gram-positive bacteria) and may not be appropriate for in vivo application. Tridecaptin M and rifampicin combination was found to be the best among all combinations and inhibited 80% of the strains tested. Moreover, the combination was stable in blood, and reasonably good therapeutic efficacy was achieved in the ex vivo blood infection model. The highest concentration of tridecaptin M used in this model was 16 µg/mL which is non-haemolytic (no significant haemolysis up to 100 µg/mL) and non-cytotoxic (IC_50_ > 250 µg/mL) [37] and feasible to achieve using intravenous administration. In an animal model, tridecaptin M was very well tolerated at a subcutaneously administered dose of 72 mg/kg. The pharmacokinetic analysis of tridecaptin is yet to be investigated. However, the study suggests that the plasma concentration of 16 µg/mL, if maintained for 4 h, could eradicate the bacterial burden significantly. Additionally, the study found that the co-administration of both compounds is not necessary. If the required concentration of both compounds can be achieved in a time lag of 2–4 h, the combination exerted the antimicrobial potential. Pre-exposure of bacteria to tridecaptin M followed by a delayed administration of rifampicin may have the same impact according to in vitro studies. This is highly advantageous in reducing the toxicity problems arising from drug–drug interactions [24]. Rifampicin showed good antimicrobial activity against MDR *A. baumannii* [28], but its monotherapy in a murine infection model leads to the rapid development of resistant mutants [25]. Rifampicin has been the most widely used antibiotic in combination with colistin or other antibiotics against *A. baumannii* in in vitro studies and in clinical trials [21,27,28,47,48,49,50]. However, these combinations were not proven statistically superior to monotherapy [11]. The main advantages of using tridecaptin M in the combination are; (1) tridecaptins do not share the mechanism of action with colistin or any of the other clinically approved antibiotics and, therefore, possess negligible chances of cross-resistance; (2) tridecaptin M showed a better safety index in an animal model in comparison to colistin [37]. Moreover, tridecaptin M, when combined with rifampicin cleared rifampicin-tolerant bacteria very effectively. In several cases, drug-tolerant bacteria or persisters are responsible for treatment failure. The combination hampered the development the persister populations significantly as compared to rifampicin alone.

To conclude, the present study offers a new opportunity to evaluate the combination of tridecaptins with rifampicin and other antibiotics to treat XDR or PDR *A. baumannii* infections. In vivo efficacy studies and pharmacokinetics are needed to develop this combination therapy further. 

## 4. Materials and Methods

### 4.1. Antibiotics, Bacterial Strains, and Growth Conditions 

Rifampicin was purchased from Sigma Aldrich, and tridecaptin M was purified from the fermentation broth of *Paenibacillus sp.* M152 as reported previously [37]. The purity of tridecaptin M was >95% as determined by HPLC. The quality control strains were purchased from HiMedia, India and *A. baumannii* ATCC 2803 was procured from ATCC, USA. Clinical isolates were obtained from Medicos Center, Chandigarh and GMCH hospital, Chandigarh, India. All the strains were maintained on Mueller–Hinton Agar (MHA) plates at 4 °C and the glycerol stocks (20%) were preserved at −80 °C. For minimum inhibitory concentration (MIC) determination and other experiments, cation-adjusted Mueller–Hinton Broth (CA-MHB) was used. For persister assays, bacteria were grown in Luria–Bertani (LB) broth. For all experiments, fresh stocks were revived each time. 

### 4.2. Outer Membrane Permeabilization Assay

A previously optimized protocol was used to assess the outer membrane permeabilization in *A. baumannii* ATCC 19606, *K. pneumoniae* ATCC 700603, and *P. aeruginosa* ATCC 27853 using NPN dye [37]. The tridecaptin M concentration was tested from 0 to 32 μg/mL. Polymyxin B was used as a positive control due to its strong membrane permeabilizing properties [51]. An NPN assay was also performed with stationary phase cells. 

### 4.3. Tridecaptin Binding Kinetics

The bacterial cultures were grown to exponential phase and centrifuged at 10,000 rpm for 8 min. The cells were washed and resuspended in PBS, and the OD_600_ was adjusted to 1.0 (~10^9^ CFU/mL). Tridecaptin M (16 μg/mL) was added to the culture (1 mL) and kept at 37 °C on shaking. At 30, 60, and 120 min, the samples were centrifuged at 13,000 rpm for 12 min and the supernatant (400 µL) was taken carefully without disturbing the pellet. The supernatant was mixed with an equal amount of 50% methanol, and the concentration of free tridecaptin M was assessed using RP-HPLC as described previously [4]. The sample without culture was taken as a negative control to calculate the area of unbound tridecaptin M and processed in similar conditions. The area under the peak for tridecaptin M was calculated and the data were plotted as the percentage of the residual drug. The negative control was considered as 100%.

### 4.4. MIC Determination 

MICs of different antibiotics were determined by micro broth dilution method as described by the Clinical and Laboratory Standards Institute (CLSI) guidelines [52]. The bacterial cell density (OD_600_) was adjusted to 0.5–0.6 and diluted 1000 times to obtain ~5 × 10^5^ CFU/mL. The antibiotics were diluted in the wells of a 96-well plate with 2-fold serial dilution. Then 100 µL of the culture was added to all the wells (total reaction volume 200 µL), and the plate was incubated for 16–18 h at 37 °C. The lowest concentration with no visible growth was recorded as MIC. 

### 4.5. Synergy Studies

The synergy of tridecaptin M with other antibiotics was initially identified by checkerboard assay [21,53]. Briefly, in a 96-well plate, the concentration of tridecaptin M was varied vertically, whereas the antibiotics were varied horizontally in a matrix of 8 × 12. The tridecaptin M concentration was tested in the range of 16–0.25 µg/mL. The concentration of antibiotics in the first well was taken as 2 × MIC, and it was 2-fold serially diluted to 512-fold in the last well. For later synergy studies, the concentration of tridecaptin was fixed to 8 µg/mL and 16 µg/mL, and the antibiotics’ concentrations were varied. The inoculum was prepared in a similar way as for MIC determination, and the plates were kept at 37 °C for MIC observation in combination. The fractional inhibitory concentration index (FICI) was calculated for each antibiotic to identify the synergistic effect of tridecaptin M [53] using following equation:

(1) $$FICI=\frac{\mathrm{MIC}\;\mathrm{of}\;\mathrm{antibiotic}\;\mathrm{in}\;\mathrm{combination}}{\mathrm{MIC}\;\mathrm{of}\;\mathrm{antibiotic}\;\mathrm{alone}}+\frac{\mathrm{MIC}\;\mathrm{of}\;\mathrm{Tridecaptin}\;M\;\mathrm{in}\;\mathrm{combination}}{\mathrm{MIC}\;\mathrm{of}\;\mathrm{tridecaptin}\;M\;\mathrm{alone}}$$

A FICI value of ≤0.5 was interpreted as synergy, whereas a value between 0.5 and 4 was interpreted as indifference and a value >4 as antagonism.

### 4.6. Time-Dependent Killing Kinetics 

*A. baumannii* ATCC 19606 was grown in CA-MHB to exponential phase, and the cell density was adjusted to ~5 × 10^5^ CFU/mL. The cells were then incubated with rifampicin (4 µg/mL) or tridecaptin M (8 µg/mL) alone as well as in combination at 37 °C and 200 rpm. The culture without any antibiotic was taken as control. At different time intervals, 20 μL of the sample, after appropriate dilution, was spotted on a MacConkey Agar plate in triplicate. The colonies were counted, and the cell viability was calculated as CFU/mL and plotted against time. 

### 4.7. Ex Vivo Blood Infection Model

Fresh rabbit blood was used to determine the potential of rifampicin and tridecaptin combination in reducing the bacterial load. Rabbit blood was diluted with an equal volume of PBS and 1 mL blood was inoculated with *A. baumannii* cells to achieve the final bacterial load of ~5 × 10^8^ CFU/mL. The blood was then incubated with either antibiotics alone or in combination. After 4 h of treatment, the samples were appropriately diluted and spread-plated on MacConkey Agar plates for colonies development. The reduction in CFU/mL of the blood was calculated.

Rabbit blood was used for in vitro infection model. The procedures for blood sampling were conducted in compliance with the ethical standards of the Institutional Animal Ethics Committee (IAEC) of the Institute of Microbial Technology (Approval no. IAEC/17/11).

### 4.8. Time-Lag Treatment Studies

*A. baumannii* ATCC 19606 was grown to late exponential phase, and OD_600_ was adjusted between 1.0 and 1.5 (10^9^–10^10^ CFU/mL). Three different experiments were performed to study the effect of time-lag between two antibiotics on bacterial killing. In the first experiment, the culture was treated for 4 h with antibiotics alone or in combination. In combination, both antibiotics were administered together. After treatment, the sample was diluted and spotted on agar plates for CFU determination. In the second experiment, the culture was pretreated with rifampicin (5 × MIC) for 4 h, and subsequently, tridecaptin M was added at two different concentrations, i.e., 8 μg/mL and 16 μg/mL. At 2 and 4 h after addition of tridecaptin M, the sample was taken and spotted on agar plates for CFU/mL measurement. In the third experiment, the samples were pretreated with tridecaptin M for 2 or 4 h. The samples were centrifuged to remove the tridecaptin M and resuspended in fresh medium containing rifampicin at 5 × MIC concentration. At 4 h after the rifampicin treatment, CFU/mL was determined as described above. Culture without any drug was taken as control.

### 4.9. Persister Assay

For persister development, previously described protocols were adopted [43,54]. *A. baumannii* strains were revived from fresh stocks on LB agar plates. Two to three colonies were inoculated in LB medium and grown to the exponential phase. The culture was diluted to 1:100 in fresh LB medium (5 mL in 50 mL falcon tubes) and incubated at 37 °C and 225 rpm for 16–18 h to reach the stationary phase. The cultures were then treated with rifampicin alone (5 × MIC) and in combination with tridecaptin M (8 μg/mL and 16 μg/mL) for 4.5–5 h. Post treatment, the samples were taken, and 20 μL was spotted on MacConkey agar plates after appropriate dilution. Persister survival was calculated by dividing the CFU obtained after treatment by the initial CFU. To confirm that the cell population consisted of persisters and was not resistant to the antibiotic, two representatives from each condition were grown in antibiotic-free medium, and their susceptibility was checked towards rifampicin as well as its combination with tridecaptin M. 

## Figures and Tables

**Table 1:** Synergetic activity of rifampicin and vancomycin with tridecaptin M against Gram-negative bacteria.

Strain	Tri-M MIC (µg/mL)	Antibiotic	Tri-M Concentration (µg/mL)	MIC (µg/mL)	Potentiation Factor	FICI
***A. baumannii*** **ATCC 19606**	32	Vancomycin	0	128		
			2	128	1	-
			4	32	4	0.375
			8	8	16	0.312
		Rifampicin	0	4		
			2	2	2	0.562
			4	1	4	0.375
			8	0.25	16	0.312
***K. pneumoniae*** **ATCC 700603**	4	Vancomycin	0	128		
			0.25	128	1	-
			0.5	128	1	-
			1.0	128	1	-
		Rifampicin	0	32		
			0.25	32	1	-
			0.5	32	1	-
			1.0	32	1	-
***P. aeruginosa*** **ATCC 27853**	256	Vancomycin	0	256		
			4	256	1	-
			8	256	1	-
			16	256	1	-
		Rifampicin	0	64		
			4	32	2	0.515
			8	32	2	0.531
			16	32	2	0.562

Tri-M—tridecaptin M; FICI—fractional inhibitory concentration index. FICI of ≤0.5 was considered as synergy. (-) represents no change in MIC, and hence, FICI was not calculated for these.

**Table 2:** Potentiation of various antibiotics in the presence of tridecaptin M in different *A. baumannii* strains.

Strain	Antibiotic	MIC (µg/mL) at Tridecaptin Concentration (µg/mL)			Fold Reduction in MIC at Tri-M (8 µg/mL)	FICI at Tri-M (8 µg/mL)
		0	4	8		
***A. baumannii*** **ATCC 19606**	Rifampicin	4	1	0.25	16	0.31
	Vancomycin	128	32	8	16	0.31
	Clarithromycin	64	16	4	16	0.31
	Imipenem	128	16	16	8	0.37
	Ceftazidime	32	8	1	32	0.28
***A. baumannii*** **ATCC 2803**	Rifampicin	256	64	64	4	0.5
	Vancomycin	64	64	16	4	0.5
	Clarithromycin	64	64	64	1	-
	Imipenem	256	256	256	1	-
	Ceftazidime	128	16	8	16	0.31
***A. baumannii*** **AB1**	Rifampicin	8	0.5	0.125	64	0.27
	Vancomycin	256	32	16	16	0.31
	Clarithromycin	128	128	128	1	-
	Imipenem	256	256	256	1	-
	Ceftazidime	128	128	128	1	-
***A. baumannii*** **AB2**	Rifampicin	256	16	8	32	0.28
	Vancomycin	256	32	16	16	0.31
	Clarithromycin	128	128	128	1	-
	Imipenem	256	125	256	1	-
	Ceftazidime	128	128	128	1	-
***A. baumannii*** **GMCH05**	Rifampicin	8	0.125	0.03	256	0.25
	Vancomycin	256	8	8	32	0.28
	Clarithromycin	128	128	128	1	-
	Imipenem	256	128	128	2	-
	Ceftazidime	128	4	4	32	0.28
